# What does media use reveal about personality and mental health? An exploratory investigation among German students

**DOI:** 10.1371/journal.pone.0191810

**Published:** 2018-01-25

**Authors:** Julia Brailovskaia, Jürgen Margraf

**Affiliations:** Mental Health Research and Treatment Center, Ruhr-Universität Bochum, Bochum, Germany; Tilburg University, NETHERLANDS

## Abstract

The present study aimed to investigate the relationship between personality traits, mental health variables and media use among German students. The data of 633 participants were collected. Results indicate a positive association between general Internet use, general use of social platforms and Facebook use, on the one hand, and self-esteem, extraversion, narcissism, life satisfaction, social support and resilience, on the other hand. Use of computer games was found to be negatively related to these personality and mental health variables. The use of platforms that focus more on written interaction (Twitter, Tumblr) was assumed to be negatively associated with positive mental health variables and significantly positively with depression, anxiety, and stress symptoms. In contrast, Instagram use, which focuses more on photo-sharing, correlated positively with positive mental health variables. Possible practical implications of the present results for mental health, as well as the limitations of the present work are discussed.

## Introduction

In 1985, Neil Postman warned his readers against a society ruled by the entertainment industry where picture media (television) would become more important than printed media [1]. Since then, the world has experienced several technological revolutions. Computers, notebooks, and mobile phones enable users to join the world of the Internet with new opportunities and dimensions in information acquisition, social interaction, and self-presentation. Fifteen years after Postman’s warning, Howard, Rainie, and Jones [2] analyzed differences in Internet using behavior and described “netizens”, people who use the Internet daily intensively for work and home life and thereby also enhance their social relationships. In year 2000, 8% of the U.S. adult population belonged into the category of “netizens”. Howard, Rainie, and Jones [2] also found that women write more e-mails than men, and that younger people use the Internet more frequently to communicate for fun via chat rooms than older people.

During the last seventeen years after this investigation, the online world has changed drastically. In June 2017, over 50% of the world population was connected to the Internet (http://www.internetworldstats.com/stats.htm). In Germany, 85% of the households use the Internet daily. In the student population, daily Internet use was reported for 100% of students [3]. Social networking sites (SNSs) belong to the most popular platforms on the Internet [4]. For example, in November 2017, the global SNS Facebook reported a total of almost 2.1 billion members and 1.37 billion daily users [5].

The popularity of SNSs has attracted the attention of researchers who have particularly focused on the relationship of personality traits and online behavior in cross-sectional studies reporting in general weak to moderate results. They described positive associations of the traits extraversion and openness with the use of SNSs, for example, time spent on SNSs and number of online-friends [4, 6–8]. In contrast, agreeableness and conscientiousness were negatively related to SNSs use [9, 10].

Cross-sectional investigations of the relationship between users’ self-esteem and SNSs use have shown inconsistent results. In studies on the use of different platforms in Germany, Poland and China, users with high self-esteem had more online-friends, commented status updates more frequently, and joined more discussion groups [10–12]. However, two U.S. studies described a negative relationship between self-esteem and the time spent on Facebook [13, 14]. Furthermore, SNSs use was shown to have greater personal importance and benefit for shy people with low self-esteem than for other users. Platforms such as Facebook enable them to build up their social capital, to acquire social support and to enhance their self-esteem [12, 15–17].

Numerous cross-sectional studies have investigated the relationship between the personality trait narcissism and SNSs use. Most of them found that different online activities, for example positing updates and photos, are positively associated with narcissism (see [18]). This result is relatively stable regardless of the investigated SNS (e.g., StudiVZ in Germany [19, 20], Renren in China [11], Facebook in the United States or other countries [20–22]) or the year of the investigation [18]. Cross-culturally, narcissistic users showed higher values of social interaction and self-presentation on Facebook than users with lower narcissism values. They had more online-friends and self-presenting pictures on their Facebook pages than other users [13, 20, 23].

In recent years, some studies have also focused on the relationship between SNSs use and mental health variables with partly contradictory results. Mostly, the strength of these results was again weak to moderate. In a cross-sectional study, Brailovskaia and Margraf [24] compared Facebook users and those not using Facebook, showing significant differences. While Facebook users had higher values of narcissism, extraversion, self-esteem, life satisfaction, happiness and social support, non-users showed higher depression symptoms. Positive mental health variables like life satisfaction and happiness were positively related to SNSs use, for example, participation in discussion groups [25–27]. The promotion of self-esteem by positive feedback from other users was discussed to have a positive influence on individual well-being [16]. Furthermore, social interaction, for example, writing and commenting status updates, was positively related to social support [27, 28].

Other studies described a negative relationship between Internet and SNSs use and mental health [29]. Kross et al. [30] found a negative association between intensive long-term Facebook use and life satisfaction in a longitudinal investigation. Cross-sectional, a positive relationship between the time spent on SNSs and depression symptoms was described [31]. Furthermore, the number of Facebook-friends was positively related to the Narcissistic and the Histrionic Personality Disorder [32].

To sum up, current mostly cross-sectional correlational results, which in general range between r = .05 and r = .35, show that SNSs use is related to personality traits as well as to various mental health variables. However, so far, final conclusions have not been made about the question whether media use can contribute to the improvement of mental health or whether it is rather a risk factor for mental health. Furthermore, it is still unclear how the individual manifestation of different personality traits can contribute to the relationship between media use and mental health.

One of the reasons for this gap in research is the fact that most of the available studies focused only on the relationship between use of SNSs and personality or mental health, neglecting the close interrelationship between the two. For example, self-esteem and extraversion have been found to be negatively related to depression symptoms (e.g., [24, 33]). In contrast, neuroticism was positively associated with negative mental health variables (e.g., [34]). Furthermore, studies considering the relationship between SNSs use and mental health often investigated either only positive (e.g., life satisfaction) or negative variables (e.g., depression symptoms). However, following the dual-factor model of general mental health, which emphasizes that mental health consists two interrelated but separate unipolar dimensions (positive mental health and negative mental health or mental illness) (e.g., [35]), it would be beneficial to investigate both dimensions together. A fully mentally healthy person is proposed to have both, a high level of positive and a low level of negative mental health [36]. An investigation including personality traits and positive as well as negative mental health variables would help to clarify earlier inconsistencies and contribute to a better understanding of the “individual–SNSs use” interaction.

Thus, considering the high importance of media use, especially Internet and SNSs use in the daily life of many people and the obvious but partly inconsistent relationships between media use, personality and mental health, the aim of the present study was to investigate these associations in more depth in a German student sample. Therefore, we included various personality traits (i.e., self-esteem, narcissism, the “Big Five”) and mental health variables (positive mental health: life satisfaction, happiness, social support, resilience; negative mental health: depression, anxiety, and stress symptoms) in our investigation.

We laid our focus on the general use of SNSs, but also wanted to shed light on the use of other media–general Internet use and computer gaming behavior. Earlier research found a positive relationship between excessive playing of computer and Internet games, and loneliness, aggression, neuroticism, depression and anxiety symptoms. Gaming behavior was negatively associated with extraversion, self-esteem, conscientiousness and life satisfaction [37–39]. Furthermore, we investigated whether different SNSs would show the same pattern of association with personality and mental health. While most of the earlier studies have been conducted on Facebook, platforms such as Twitter, Tumblr and Instagram have gained only little attention in research. Results found on Facebook are often treated as universally valid and applicable to all SNSs. However, it is important to bear in mind that while all social platforms base on the same idea defined by Boyd and Ellison [40]–to construct a profile of the own person and to build relationships with other users–each SNS has its own characteristics. For example, on Instagram the main focus is laid on interaction through photo-sharing, whereas on Twitter more attention is laid on users’ blogging behavior. Thus, the question arises, whether the results concerning Facebook can be generalized to other SNSs or whether the specific characteristics of the individual SNSs contribute to differences in the result pattern. Two cross-sectional studies investigating both Twitter use and Facebook use reported both to be positively associated with the trait narcissism. But when considering specific facets of narcissism, the association patterns partly differed between the two SNSs [41, 42].

Due to the partly inconsistent or missing findings of earlier research, the present study had an exploratory character. However, in order to obtain an overall picture, we focused on several general research questions including different variables of media use, personality, and mental health:

How are the personality traits and mental health (positive and negative) variables related to computer gaming behavior? (Research Question 1)

How are the personality traits and mental health (positive and negative) variables related to general Internet use? (Research Question 2)

How are the personality traits and mental health (positive and negative) variables related to general SNSs use? (Research Question 3)

Does membership on the SNSs Facebook, Twitter, Tumblr and Instagram show the same association patterns with the personality traits and mental health (positive and negative) variables? (Research Question 4)

Considering the strength of associations demonstrated by earlier studies in this research field, we expected to find weak/small to moderate effects.

The present exploratory study is part of the ongoing BOOM (Bochum Optimism and Mental Health) research program. In various studies, the BOOM program investigates risk and protective factors of mental health [43, 44].

## Materials and methods

### Procedure and participants

The present sample included 633 freshmen of a large German university (419 women, 214 men; age in years: M = 21.80, SD = 5.35, range: 16–59). While 52.9% of the participants were single, 43.4% lived in a steady relationship and 3.6% were married. To collect data, a collective e-mail including a participation invitation and an online-link of the German language self-report survey (research platform www.unipark.de) was sent to a group of 749 freshmen of the university at the beginning of the winter semester 2016/2017, who allowed to contact them for study participation. Thus, the response rate was 84.5%. While 110 of the non-responders did not start the online survey, six persons terminated it after the first few questions. Therefore, it was not possible to analyze their data adequately. Data collection continued from October to December 2016. Participation was voluntary and could be compensated by course credits. A priori conducted power analyses (G*Power) showed that our sample was large enough to reach valid results (power > .80). We received research and Ethics Committee approval of the Ethics Committee of the Faculty of Psychology of the Ruhr-Universität Bochum for the implementation of the present study. All national regulations and laws regarding human subjects research were followed. Participants were properly instructed and gave online informed consent to participate. This work was supported by the Alexander von Humboldt foundation. The dataset used in the present study is available in S1 Dataset.

### Measures

#### Media use

Participants were asked about their media use habits on 7-point Likert scales (0 = never, 1 = less than once a month, 2 = once or twice a month, 3 = once a week, 4 = several times a week, 5 = once a day, 6 = more than once a day): playing computer games (“How often do you play computer and Internet games (excluding games on SNSs)?”), general Internet use (“How often do you use the Internet as a whole?”), and SNSs use (“How often do you use social platforms?”). Participants who used SNSs were asked whether they are members of the SNSs Facebook, Twitter, Instagram, Tumblr (0 = no, 1 = yes), or any other platform (“If you use any other platforms, name them please.”).

#### Personality traits

Narcissism. The personality trait narcissism was assessed with the brief Narcissistic Personality Inventory (NPI-13) [45], which showed similar good psychometric properties as the full-length NPI-40 [46, 47]. This instrument included 13 items rated in a forced-choice format (0 = low narcissism value, 1 = high narcissism value; original scale reliability: Cronbach’s α = .82). Similar to earlier studies that reported the NPI-13 to have an internal scale reliability of Cronbach’s α = .67 [47], current internal reliability was α = .65.

Self-esteem. The global self-esteem was measured with the Single-Item Self-Esteem Scale (SISE) [33]. Earlier research [48] found this instrument to be equally valid and reliable as the Rosenberg Self-Esteem Scale [49]. It consisted of the statement “I have high self-esteem.” rated on a 5-point Likert scale (1 = not very true of me, 5 = very true of me; mean across-time correlation: r_m_ = .75).

“Big Five”. To measure the “Big Five” personality traits, we used the Big Five Inventory 10 (BFI-10) [50]. It included ten items rated on a 5-point Likert scale (1 = disagree strongly, 5 = agree strongly). Considering that each of the five scales included only two items, to investigate the scale reliability of the BFI, we followed the recommendation of Clark and Watson [51] by calculating the mean interitem correlation (r_mi_) additionally to the Cronbach’s α for each scale: extraversion (α = .78, r_mi_ = .63), agreeableness (α = .45, r_mi_ = .29), conscientiousness (α = .40, r_mi_ = .26), neuroticism (α = .55, r_mi_ = .38), and openness (α = .54, r_mi_ = .38).

#### Mental health

Life Satisfaction. The Satisfaction with Life Scale (SWLS) [52, 53] measured life satisfaction with five items which were rated on a 7-point Likert scale (1 = strongly disagree, 7 = strongly agree). Current scale reliability was α = .86.

Happiness. To assess happiness, we used the Subjective Happiness Scale (SHS) [54]. Participants rated four items on a 7-point Likert scale (range: 1–7). Current scale reliability of the SHS was α = .86.

Resilience. Resilience was measured with the German Resilience Scale (RS-11) [55, 56]. The 11 items of this instrument were rated on a 7-point Likert scale (1 = disagree, 7 = agree). Current internal reliability was α = .86.

Social Support. To investigate subjective perceived or anticipated social support, the short version of the Questionnaire Social Support (F-SozU K-14) [57] was used. The 14 items were rated on a 5-point Likert scale (1 = not true at all, 5 = very true). Current internal reliability was α = .93.

Depression, Anxiety, Stress. Depression, anxiety, and stress symptoms over the previous week were measured with the Depression Anxiety Stress Scales 21 (DASS-21) [58], a well-established instrument in non-clinical and clinical samples. The DASS-21 consisted of three 7-item subscales which we found to have different high scale reliability (depression: α = .91, anxiety: α = .79, stress: α = .86) rated on a 4-point Likert scale (0 = did not apply to me at all, 3 = applied to me very much or most of the time) [59, 60].

The German versions of all instruments measuring personality traits and mental health variables (positive and negative) have been validated earlier in the framework of the BOOM research program (e.g., [24, 47]).

### Statistical analyses

Statistical analyses were conducted with the Statistical Package for the Social Sciences (SPSS) 24. After the descriptive analyses of the investigated variables, their mutual associations were assessed by zero-order bivariate correlations and three multiple hierarchical regression analyses. The regression analyses included, respectively, depression, anxiety, and stress symptoms as the dependent variable. The variables age and gender were added in the first step, the personality traits were added in the second step, the positive mental health were added in the third step, and SNSs use was added in the fourth step of the analyses.

## Results

### Descriptive analyses

All investigated psychological variables were close to normally distributed (indicated by Kolmogorov-Smirnov test, analyses of skew, kurtosis, and histogram). The descriptive values of media use, personality traits, and mental health variables are presented in Table 1 and Table 2.

**Table 1:** Frequency (%) of media use.

	Computer games	Internet	SNSs
(0) “never”	34.8	0	8.1
(1) “less than once a month”	22.4	1.1	10.4
(2) “once or twice a month”	7.9	0.5	9.3
(3) “once a week”	7	2.7	9.6
(4) “several times a week”	11.2	5.7	17.1
(5) “once a day”	6.5	17.7	17.2
(6) “more than once a day”	10.3	72.4	28.3

N = 633; SNSs = social networking sites; due to rounding, the sum of listed figures may be higher/lower than 100%.

**Table 2:** Means, standard deviations, minima, and maxima of personality traits and mental health variables.

	M	SD	Min	Max
**Personality traits**				
NP-13	4.06	2.53	0	13
SISE	3.31	1.06	1	5
BFI-10: Extraversion	6.26	2.15	2	10
BFI-10: Agreeableness	7.09	1.61	2	10
BFI-10: Conscientiousness	6.72	1.68	2	10
BFI-10: Neuroticism	6.33	1.95	2	10
BFI-10: Openness	7.42	2.01	2	10
**Mental health**				
SWLS	24.29	6.16	5	35
SHS	18.17	5.48	4	28
RS-11	58.05	9.42	11	77
F-SozU	58.66	10.09	14	70
DASS: Depression	5.30	5.10	0	21
DASS: Anxiety	4.15	4.00	0	20
DASS: Stress	7.12	4.76	0	21

N = 633; M = Mean; SD = Standard Deviation; Min = Minimum; Max = Maximum; NPI = Narcissistic Personality Inventory; SISE = Single-Item Self-Esteem Scale; BFI = Big Five Inventory; SWLS = Satisfaction with Life Scale; SHS = Subjective Happiness Scale; RS = Resilience Scale; F-SozU = Questionnaire Social Support; DASS = Depression Anxiety Stress Scales.

On average, the participants used 1.63 SNSs (SD = 1.00; range: 0–6; no SNSs: 8.1%; one: 43.6%; two: 31.4%; three: 12.6%; four: 3.2%; five: 0.8%; six: 0.3%). Facebook was used by 76.3% of the participants, Instagram by 39.7%, Twitter by 10%, Tumblr by 8.7%, and 24.8% used further SNSs such as Xing and LinkedIn.

### Associations between media use, personality and mental health

Table 3 and Table 4 show the relationships between media use, personality, and mental health variables. Playing computer games was significantly negatively related to mostly all investigated psychological variables. However, its relationship with depression symptoms was significantly positive. While Internet use correlated significantly positively with narcissism, self-esteem, and the positive mental health variables, its correlation with agreeableness was significantly negative. SNSs use was (marginally) significantly positively related to narcissism, self-esteem, extraversion, life satisfaction and social support. Its associations with the negative mental health variables did not become significant.

**Table 3:** Correlations of personality traits and media use variables.

	NPI-13	SISE	BFI: Extraversion	BFI: Agreeableness	BFI: Conscientiousness	BFI: Neuroticism	BFI: Openness
**Use frequency**							
Computer games	.04	-.08*	-.22**	-.14**	-.27**	-.12**	.01
Internet	.11**	.10*	.05	-.12**	-.04	-.06	-.01
SNSs	.15**	.08*	.16**	-.05	-.04	.01	-.04
**Member (no; yes)**							
Facebook	.11**	.09*	.24**	.01	.01	-.04	-.07^(^*^)^
Twitter	-.07	-.08*	-.08*	-.11**	-.14**	-.01	.02
Instagram	.04	.04	.17**	.05	-.07^(^*^)^	-.02	.01
Tumblr	-.05	-.09*	-.06	.05	-.08^(^*^)^	.07^(^*^)^	.11**
SNSs number	.04	-.02	.15**	-.01	-.11**	.02	.03

N = 633; NPI = Narcissistic Personality Inventory; SISE = Single-Item Self-Esteem Scale; BFI = Big Five Inventory; SNSs = Social Networking Sites.

**p < .01

*p < .05

(*)p < .10.

**Table 4:** Correlations of mental health variables and media use variables.

	SWLS	SHS	RS-11	F-SozU	DASS: Depression	DASS: Anxiety	DASS: Stress
**Use frequency**							
Computer games	-.08*	-.12**	-.08*	-.18**	.10**	.03	-.09*
Internet	.09*	.05	.13**	.13**	-.03	-.04	-.01
SNSs	.08^(^*^)^	.06	.05	.13**	.03	-.01	.06
**Member (no; yes)**							
Facebook	.11**	.06	.09*	.11**	-.05	-.06	.01
Twitter	-.15**	-.12**	-.12**	-.13**	.12**	.11**	.02
Instagram	.09*	.07	.02	.11**	-.01	.02	.01
Tumblr	-.10**	-.10*	-.11**	-.10*	.08*	.15**	.13**
SNSs number	.02	-.01	-.02	.05	.04	.07^(^*^)^	.06

N = 633; SWLS = Satisfaction with Life Scale; SHS = Subjective Happiness Scale; RS = Resilience Scale; F-SozU = Questionnaire Social Support; DASS = Depression Anxiety Stress Scales; SNSs = Social Networking Sites.

**p < .01

*p < .05

(*)p < .10.

Number of SNSs correlated (marginally) significantly positively with extraversion and anxiety, and negatively with conscientiousness. While membership on Facebook was significantly positively related to narcissism, self-esteem, extraversion and most of the positive variables, its association with openness was marginally significantly negative. Twitter membership correlated significantly negatively with different personality traits and all positive mental health variables. Its correlation with depression and anxiety symptoms was significantly positive. A significant positive relationship was also found between Instagram membership and extraversion, life satisfaction, and social support. The association between Instagram membership and conscientiousness was marginally significantly negative. Tumblr membership correlated (marginally) significantly negatively with self-esteem and conscientiousness, and (marginally) significantly positively with neuroticism and openness. Its correlation pattern with the mental health variables resembled the pattern of Twitter membership.

Next, the associations of the negative mental health variables with personality, positive mental health variables and SNSs use, the variable which our main focus was laid on, were analyzed further by calculating three multiple regression analyses. In all three analyses, there was no violation of multicollinearity assumption as all values of tolerance were > .25, and all variance inflation factor values were < 5 (see [61]).

While in the first model with depression symptoms as the dependent variable, SNSs use showed a very weak but significant result (SNSs use: changes in R^2^ = .004, F(1,618) = 4.551, p = .033; standardized beta = .066, p = .033, 95% CI [.013;.323]), in the model with anxiety symptoms as dependent variable (SNSs use: changes in R^2^ = .000, F(1,618) = .015, p = .903; standardized beta = .004, p = .903, 95% CI [-.131;.148]), as well as in the model with stress symptoms as dependent variable (SNSs use: changes in R^2^ = .002, F(1,618) = 2.241, p = .135; standardized beta = .050, p = .135, 95% CI [-.037;.276]) SNSs use did not become significant. Thus, while the independent predictive value of SNSs use was 0.4% for depression symptoms, it did not independently predict anxiety and stress symptoms.

## Discussion

The present study investigated the relationship between media use, particularly SNSs use, personality and mental health (positive and negative variables), showing weak but significant results. To the best to our knowledge, this is the first study in this context that takes four different SNSs into consideration.

Our first research question referred to the relationship between computer gaming behavior, personality and mental health (see Research Question 1). In accordance with earlier cross-sectional research [39], we found a negative association between the frequency of playing computer games and the positive mental health variables life satisfaction, happiness, resilience and social support. Furthermore, depression symptoms were positively related to the gaming frequency. However, participants who played computer games more frequently perceived fewer stress symptoms. One possible explanation for this finding is that frequent gamers use their gaming behavior as a stress coping strategy, which, however, not only buffers stress, but also negatively affects their well-being. Thus, our findings showed that it is reasonable to investigate positive and negative mental health variables separately, corresponding to the dual-factor model of mental health which defines mental health on two interrelated but separated unipolar dimensions (positive and negative) [62, 63].

Participants who frequently used the Internet seemed to have higher values of self-esteem, narcissism, life satisfaction, resilience and social support than others (see Research Question 2). The following considerations could partly explain this result. Narcissism and self-esteem were significantly positively interrelated (r = .34, p < .01) and both correlated positively with the positive mental health variables life satisfaction (narcissism: r = .18, self-esteem: r = .56, both: p < .01), resilience (narcissism: r = .21, self-esteem: r = .55, both: p < .01) and social support (narcissism: r = .09, p < .05, self-esteem: r = .38, p < .01). The increase of self-esteem belongs to the main aims of narcissistic people [64]. To achieve this aim, they frequently selectively search for information which confirms their grandiosity and fade out all information which contradicts this image [65]. When narcissistic people find confirmation for their uniqueness and superiority, their self-esteem increases, and they feel satisfied with their life [66]. We asked our participants about their general Internet use, including also, for example, writing e-mails, taking part in group discussions and blogging. Probably, narcissistic people who frequently talk online about themselves and their concerns, also receive frequent feedback from other users. Since they selectively search for positive feedback, they perceive a high amount of social support [57]. Further, social support and resilience were significantly positively interrelated (r = .53, p < .01), and earlier studies have found them to influence the development of one another [67–69]. Thus, online experience of social support could increase users’ resilience, making them more resistant to negative feedback.

Disregarding the relationship between personality and mental health, it should be mentioned that the Internet also offers many services such as online banking and shopping, that facilitate everyday tasks, which also can contribute to life satisfaction of its users.

Furthermore, general Internet use was negatively associated with agreeableness. Many online-activities remain anonymous, for example, the participation in forum-discussions. Anonymity can increase the probability of writing negative comments even though such comments could hurt someone’s feelings.

We also focused on general SNSs use (see Research Question 3). Present results show that it could make a difference whether the focus of the investigation is on the number of used SNSs or on the frequency of their use. Extraverted as well as anxious users seemed to prefer to join more platforms than others. Extraverted people are sociable and search for new interaction partners [70]. For shy and socially anxious people, SNSs provide an appropriate forum to enhance their social capital [17]. Thus, the higher the number of SNSs memberships by extraverted or anxious users, the higher is the possibility that they find more interaction partners. Furthermore, extraverted participants not only used more SNSs than others, they also used these SNSs more frequently. However, this was not the case for users with higher anxiety values. The personality traits narcissism and self-esteem were positively related to SNSs use frequency. Narcissistic people search for attention and admiration which increase their self-esteem [66]. To achieve their aim, they tend to invest a lot of time in frequent online self-presentation and social interaction [20].

Earlier cross-sectional studies reported positive associations between SNSs use and depression symptoms [31]. For example, time spent on Facebook was positively associated with depression [31, 71]. Frequent and long-lasting Facebook interaction was related to greater distress [72] and negative self-evaluation [73] which increase depression symptoms. Interestingly, in our present study, SNSs use independently explained only 0.4% of the variance of depression symptoms over and above all the other investigated variables. On a bi-variate level, there was no significant relationship between SNSs use and depression symptoms. Furthermore, SNSs use did not independently predict anxiety and stress symptoms over and above the other variables. One reason for the current results might be that in the present study, participants rated the frequency of their general SNSs use, while in earlier investigations use of specific SNSs and specific online activities (e.g., interaction with other platform members) have been considered. Thus, present measure could be too general to identify specific relationships with regard to negative mental health variables.

This conclusion is partly supported by the results gained from the investigation of the association pattern of individual SNSs (see Research Question 4). Studies investigating the association between SNSs use and personality or/and well-being concentrated mostly on Facebook. Our results showed that also the membership on other SNSs is related to different personality traits and mental health variables, and that the association patterns partly differ between the investigated SNSs. Similar to earlier studies, we found a positive association between narcissism, extraversion and Facebook membership [7]. Facebook belongs to the most popular SNSs and offers its members various functions for self-presentation [20]. Narcissistic and extraverted people use these possibilities to get attention, to gain new social interaction partners, and, especially narcissists, to gain popularity and admiration. In our study, Facebook membership was also positively related to self-esteem. On Facebook, users have many possibilities for engaging in social communication which contributes to building up their social capital [15] and self-esteem [74]. Thus, it cannot be ruled out that the self-esteem of our participants increased due to Facebook use. However, to verify this assumption, longitudinal studies that also measure the duration of participants’ membership and activity level are needed (see also [75]).

The positive mental health variables life satisfaction, resilience and social support were positively associated with Facebook membership. The more Facebook-friends a user has, the higher is the possibility that one of them is online, will discuss the user’s problems, share his opinions and give him positive feedback. This can enhance the user’s feeling of social support, life satisfaction and well-being [76, 77]. Such online support could probably promote user’s resilience and make him more resistant to difficulties in the offline-world.

In a cross-sectional study, Davenport, Bergman, Bergman, and Fearrington [78] found a significant positive association between Twitter use and narcissism in college students. In our also cross-sectional study, narcissism was (not significantly) negatively related to Twitter membership. Further, extraversion and self-esteem, which both are described to be important traits of narcissism [66, 79], were negatively related to Twitter membership [80]. This was also the case for agreeableness and conscientiousness. However, it should be mentioned that only 10% of our participants used Twitter. Thus, present results should be considered with caution.

Earlier studies have shown Twitter to be a popular forum for communication of (mental) health problems [81, 82]. Interestingly, we found Twitter membership to be negatively related to life satisfaction, happiness, social support and resilience, and positively associated with depression and anxiety symptoms. Therefore, the discussion of (mental) health concerns seems to attract more people with lower mental health values. Probably, they use Twitter to share their experiences regarding health problems.

Thus, while extraversion, self-esteem and positive variables were positively related to Facebook membership, their association with Twitter membership was negative. One possible explanation for this difference in the following: Typically, Twitter users express themselves mostly by short tweets that can be seen by their followers. Tweeting is more a kind of self-presentation to a large audience than a mutual exchange, even though followers can response to tweets [83]. In comparison to the more diverse interaction on Facebook, interaction on Twitter seems to be more impersonal and less likely to enhance a person’s social capital, which is important for self-esteem, life satisfaction and social support [12]. However, taking into account that only 10% of our participants used Twitter and the weak found associations, these conclusions should be considered with caution.

Comparable to the results on Twitter, we also found a negative relationship between self-esteem, the four positive mental health variables and Tumblr membership. Furthermore, depression, anxiety, and stress symptoms were positively associated with the use of this SNS.

The nature of photos posted on Instagram is related to the individual “Big Five” manifestation [84]. Our study showed a positive relationship between extraversion, life satisfaction, social support and Instagram membership. Thus, future research should investigate these associations in greater depth by analyzing also the content of the photos: Are people with more positive well-being more likely to post photos with positive content to share their well-being with others? Does a glance at positive photos posted by others enhance well-being?

Considering the results on individual SNSs, we can conclude that platforms which focus more on written interaction (Twitter, Tumblr) were rather negatively associated with positive mental health variables, while their relationship with negative variables (e.g., depression symptoms) was positive. In contrast, the membership on Instagram, which focuses on photo-sharing, was rather positively associated with positive mental health variables. This is in line with the results of a cross-sectional eye-tracking study conducted with participants aged 18–31 which showed a preference for online pages including a main large image and little text [85]. Thus, there is a need for longitudinal studies to investigate whether the use of these SNSs influences mental health or/and whether people with a certain state of mental health prefer a specific kind of SNSs.

To sum up, our results showed that there is a significant association between media use (especially SNSs), personality and mental health. However, it is not possible to make general statements about the positive or negative effects of media use on our mental health. Therefore, it is crucial to clearly differentiate between the type of media, the kind of SNSs, and the definition of mental health and its positive or negative variables.

### Limitations and further research

The present study has some limitations, which should be discussed. It is important to emphasize that similar to most studies considering media use, the present study has a cross-sectional design. For this reason, we only can make correlational conclusions and hypothesize about the direction of the found associations, such as the positive relationship between the positive mental health variables and Instagram membership. Additionally, the found associations mostly are weak. Given the fact that the strength of associations between different variables (besides autoregressive variables) normally decreases rapidly as time between the measurements of the investigated variables increases, our present results should be considered with caution and replicated in further studies. Thus, in order to make causal statements, it is necessary to expand cross-sectional research through longitudinal prospective studies which observe the longitudinal course and possible changes over time of the found associations (see [75]).

We used online self-report questionnaires to assess data, so social desirability, a well-known problem in questionnaire surveys, cannot be excluded. For future questionnaire studies, we advise to use instruments measuring the tendency of social desirability, for example, the Balanced Inventory of Desirable Responding (BIDR) [86]. Moreover, earlier studies (e.g., [87]) found discrepancies between self-reported and actual use of SNSs, such as Facebook. One reason for these discrepancies is that because of the “ubiquitous nature” [87] (p. 630) of SNSs use in daily life, users often are not able to accurately remember the amount or frequency of their actual use. Therefore, future studies should focus on actual online behavior and objective measures (e.g., data taken from the participants’ Facebook account) (see [10, 19, 20]).

Furthermore, all participants were freshmen at a German university, which limits the generalizability of our results. Cross-cultural replication with a sample with a broader age range and a similar proportion of male and female participants is desirable.

Global self-esteem was assessed by the Single-Item Self-Esteem Scale, a self-report measure which consists of only one item. Even though this instrument has been demonstrated to be a reliable and valid measure [33, 48], because of its shortness it is only a rough measure and cannot cover all facets of the corresponding construct self-esteem. Therefore, our present results and interpretations regarding self-esteem should be considered with caution. We advise further studies to replicate them by using a longer self-esteem measure, such as the Rosenberg Self-Esteem Scale [49].

Furthermore, it should be considered that the single scales of the BFI, which was used to assess the “Big Five” traits, had low reliability values–a well-known problem of brief measures [88]. Thus, future studies should replicate current results by using for example the NEO Five-Factor Inventory [70].

In the present study, participants were asked about their SNSs use frequency. However, the extent of their social interaction and self-presentation, which earlier studies showed to be associated with various personality traits [19, 20] and well-being [28], was not investigated. Considering that passive Facebook use could negatively influence mental health [89], we advise further studies to analyze the relationship between individual SNSs use variables, such as the number of online-friends, groups, photos, “Likes”, and positive and negative mental health variables in longitudinal prospective investigations. Such findings will most likely indicate how we can better protect mental health, especially the well-being of younger generations who belong to the most intensive users of SNSs [90].

## Supporting information

S1 Dataset Dataset used for analyses in present study. (SAV)
